# Translation and validation into Brazilian Portuguese of the Post-Catheterization Questionnaire, or Cooper Scale, for post-puncture quality of life analysis in diagnostic arteriography of the lower limbs

**DOI:** 10.1590/1677-5449.202400652

**Published:** 2026-03-16

**Authors:** Igor Calixto Novais Dias, Marcelo Passos Teivelis, Gabriel Grizzo Cucato, Felipe Nasser, Marcelo Calil Burihan, Henrique Jorge Guedes

**Affiliations:** 1 Faculdade Santa Marcelina, São Paulo, SP, Brasil.; 2 Faculdade Israelita de Ciências da Saúde Albert Einstein – FICSAE, São Paulo, SP, Brasil.; 3 Universidade Federal de São Paulo – UNIFESP, São Paulo, SP, Brasil.

**Keywords:** Cooper Scale, questionnaires, validation, radial, arteriography

## Abstract

**Background:**

The Cooper Scale is an English-language questionnaire specifically designed to analyze quality of life after arterial puncture for procedures such as arteriography and angioplasty. The CS also specifically asks about the patient’s preference for radial or femoral artery access.

**Objectives:**

To translate the Cooper Scale into Brazilian Portuguese and validate it.

**Methods:**

The translation process was conducted in accordance with international linguistic validation guidelines with a sample of 62 patients with peripheral arterial occlusive disease who underwent diagnostic arteriography at a quaternary hospital in São Paulo, Brazil. Reliability was assessed with tests of reproducibility (test-retest with a 24-hour interval) and internal consistency. Construct validity was analyzed in terms of the correlation between the items on the Cooper Scale and those of the EUROQOL 5D-5L, which was also administered to the 62 patients in the sample.

**Results:**

The translated Cooper Scale had moderate internal consistency and high reliability in the test-retest analysis, with intraclass correlation coefficients ranging from 0.563 to 0.834 (p < 0.001). Significant correlations were observed between the items on the Cooper Scale and those on the EQ-5D-5L.

**Conclusions:**

The Cooper Scale was translated into Brazilian Portuguese and then validated, demonstrating adequate psychometrics and reliability for administration to patients who have undergone arterial punctures.

## INTRODUCTION

Digital subtraction angiography is considered one of the most important techniques for acquisition of vascular images,^1,2^ especially of the lower limbs. Choosing the most appropriate puncture site is very important to reduce the incidence of complications and optimize procedure duration. The ideal access should be easy to find, using anatomic references or by palpation of pulses, or with the aid of methods such as radioscopy and Doppler ultrasonography.^2^ Historically, the femoral artery has been most often used for access for arteriography of the lower limbs, accounting for more than 80% of accesses for cardiac angiographs in the United States during the 2000s.^2^ However, this type of puncture requires the patient to be in bed and causes significant discomfort during compression of the puncture site, primarily during the first 24 hours after the procedure.

As technology has evolved, the devices used for these procedures have become smaller, with better navigability and torque, enabling use of the radial artery as an alternative access route. This access is associated with similar complication rates but better post-procedural quality of life for patients.^3-5^ In Japan, the radial artery is used for coronary interventions in approximately 75% of cases.^4^ Despite its smaller caliber and the greater technical difficulty of puncture, radial access offers advantages such as increased patient comfort, easier monitoring for immediate complications, early mobilization, and shorter length of hospital stay.^6^

While the radial artery can also be used for procedures targeting other arterial territories, such as lower limbs with occlusive peripheral arterial disease, there is not yet evidence that patients with this condition prefer this type of access, as is the case among those with coronary disease. In this respect, quality of life questionnaires can be of use for assessing this preference.

Considering the Brazilian scenario, in which large numbers of invasive examinations including arteriography of the lower limbs and aortography are still being conducted, use of specific instruments validated in Brazilian Portuguese can facilitate choice of the best arterial access for this subset of patients.

Thus, the objective of this study was to translate the Post-Catheterization Questionnaire, also known as the Cooper Scale (CS), into Brazilian Portuguese and validate the translated version. This instrument, first described by Cooper et al.,^6^ assesses quality of life after arterial puncture, contributing to determination of the ideal access for different groups of patients who need invasive examinations such as diagnostic arteriography.

## METHODS

The study was conducted at the Hospital Israelita Albert Einstein (HIAE), in São Paulo, Brazil, in partnership with the Hospital Santa Marcelina (HSM). The protocol was registered on the Plataforma Brasil registry (HIAE Ethics Appraisal Submission Certificate [CAAE]: 02763518.9.0000.0071; HSM CAAE: 02763518.9.3001.0066) and approved by the HIAE Research Ethics Committee (opinion number 3.241.955) and the HSM Research Ethics Committee (opinion number 3.482.845/2019).

### Translation and cultural adaptation procedures

Using protocols described elsewhere,^7-11^ the CS underwent a translation and cross-cultural adaptation process, employing the back-translation method.^7^ Initially, the first translation was conducted by an independent professional, a native speaker of Brazilian Portuguese fluent in English. The translator was not informed of the study objective and was instructed to conduct a semantic translation, rather than simply performing a literal translation.

Later, the translated version was revised by the authors in conjunction with a multidisciplinary committee comprising vascular surgeons, hemodynamic nurses, and cardiologists, all with experience in diagnostic arteriography. This team analyzed each item on the questionnaire and suggested alterations to improve the language, where relevant. During this stage, in conjunction with the committee and with the help of the translator, the authors also evaluated possible Brazilian regional linguistic variations. A second version of the instrument was developed based on these contributions.

**Figure 1:**
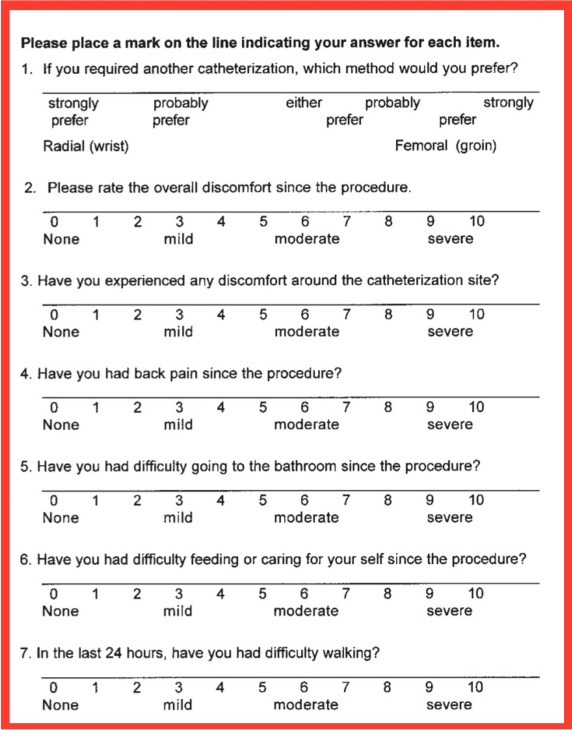
Original Cooper Scale.

**Figure 2:**
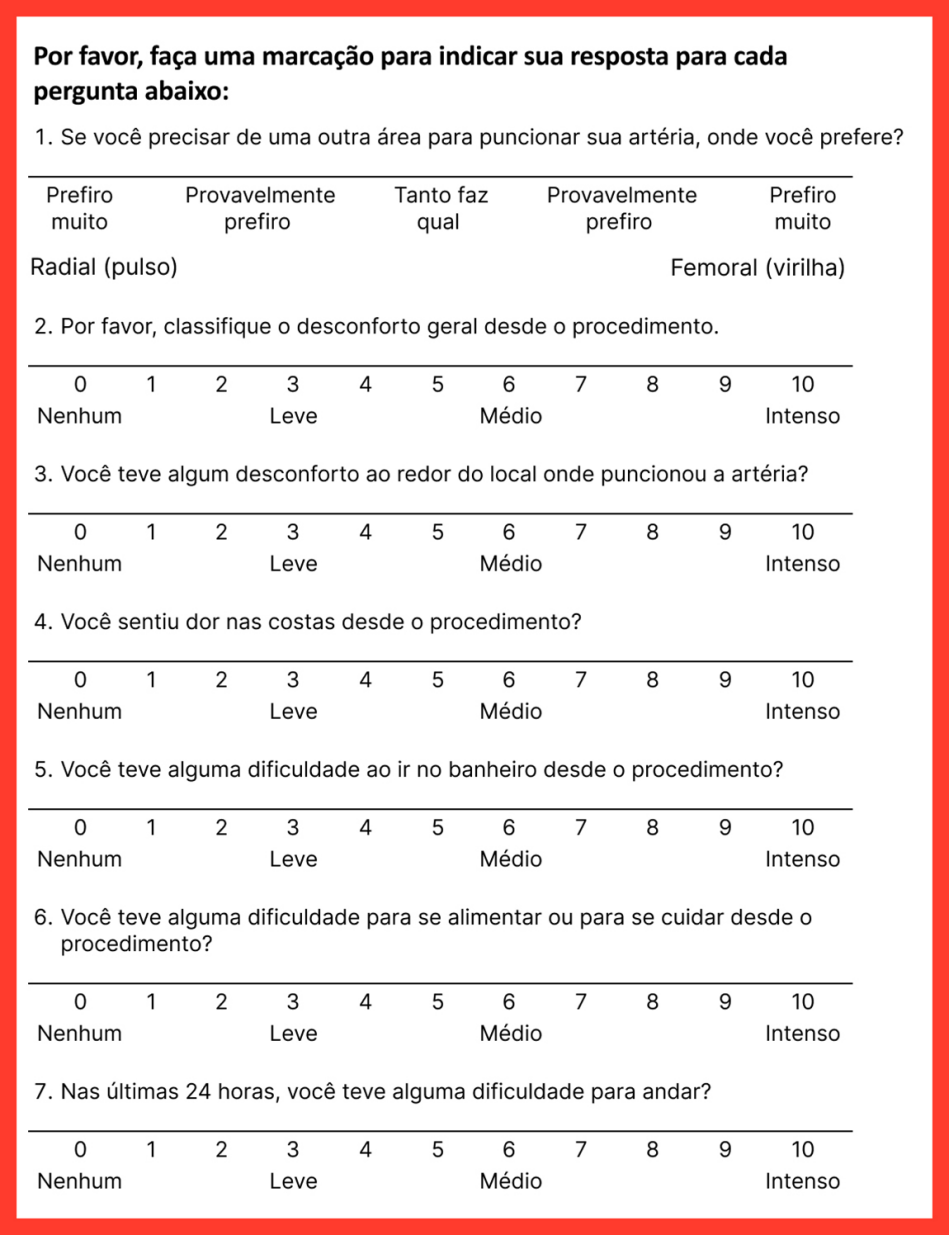
Cooper Scale –final Brazilian Portuguese version.

Next, a back-translation was conducted by a professional translator, a native speaker of English fluent in Brazilian Portuguese. This professional did not have access to the original instrument. The backtranslated version was then compared to the original version in English by the same multidisciplinary committee and by the authors, resulting in a final consensus version in Portuguese, using simple and direct vocabulary. The original version of the CS and the final version of the translation are shown in Figures 1 and 2, respectively.

### Linguistic validation

Linguistic validation is a process in which an instrument is tested with a representative sample of the target population to determine whether the interviewees understand the translated questionnaire in the same manner that the original would be understood. Following international protocols,^8-11^ first, a pilot test is conducted with three to five participants, while the subsequent stages require a sample comprising seven to ten participants per question. Since the CS contains seven questions, a minimum of 49 patients would be necessary.

**Figure 3:**
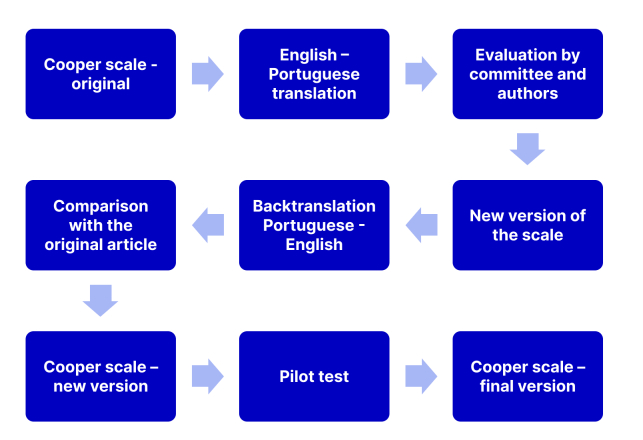
Flowchart illustrating the process of transcultural linguistic validation of the English Cooper Scale (original language) into Brazilian Portuguese (target language).

Therefore, a pilot test was conducted with five patients, who evaluated the clarity of the questionnaire and the terms employed in it, and were also asked to suggest improvements where relevant. The authors then performed a second analysis based on the participants’ suggestions. It was decided to conduct the cognitive analysis with the first 62 patients enrolled. A flowchart illustrating the process of linguistic validation is shown in Figure 3.

### Psychometric validation

Psychometric validation was conducted using the same sample of 62 patients, who completed the CS twice: once immediately after removal of the introducer sheath (T1) and again 24 hours after the procedure (T2). Different interviewers administered the questionnaire each time. Although the original protocol stipulated a third administration at a time from 7 to 14 days after T1, this step was eliminated because of losses to follow-up, caused by the COVID-19 pandemic and because hospitalized patients underwent their definitive surgical procedures soon after arterial puncture.

**Figure 4:**
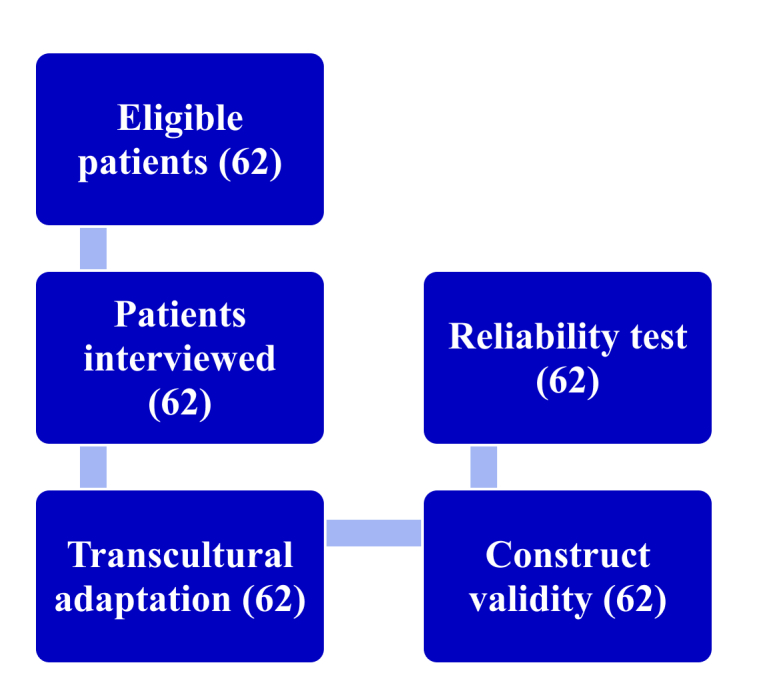
Flowchart illustrating the process of linguistic and psychometric validation of the Cooper Scale and the samples of study participants.

The reliability of the CS was assessed using tests of reproducibility (test-retest) and internal consistency.^12,13^ Construct validity was tested by comparison of the CS with the EUROQOL 5D-5L (EQL) questionnaire, which was also administered to the same 62 patients. The EQL was chosen because it is a quality-of-life questionnaire that is easy to administer and has been validated for Brazilian Portuguese.^10^ A flowchart illustrating the validation process is shown in Figure 4.

### Population and inclusion/exclusion criteria

Patients were enrolled who had been scheduled for diagnostic arteriography of the lower limbs at a quaternary hospital from 2020 to 2023. All were given and signed a free and informed consent form before the procedure, which had been standardized in advance by the institution and covered all the technical terms used in the questionnaire. These terms were also explained to patients by the medical team during administration of the free and informed consent form. In response to the COVID-19 pandemic, the original study design, which had envisaged enrollment of outpatients only, was altered to also include hospitalized patients. All of the patients agreed to take part in the study.

The exclusion criteria were patients with total loss of waveform in the Barbeau test (type D),^4^ with a failed catheterization attempt via any access route, presence of ulnar or femoral artery occlusion, known coagulopathies, radial artery with diameter less than 2 mm, and illiteracy.

All examinations were conducted by physicians with experience with both access routes, following international protocols.^14,15^ Hemostasis was obtained by manual compression for 15 minutes, followed by a compressive dressing, for 6 hours for the femoral artery or 4 hours for the radial artery.

### Randomization

**Figure 5:**
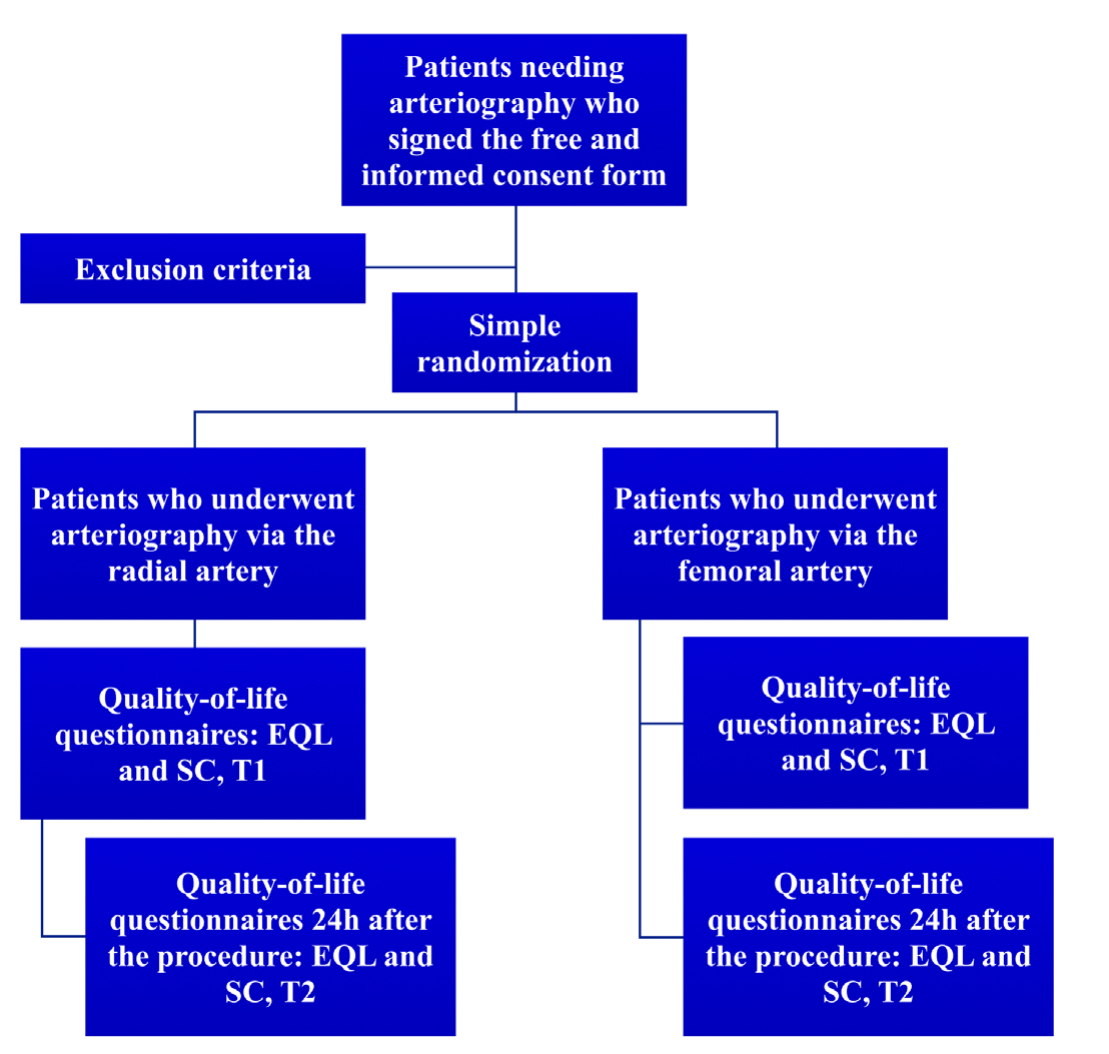
Flowchart illustrating the process of patient recruitment for data analysis and the times of administration of the questionnaires. EQL = EUROQOL 5D-5L; CS = Cooper Scale; T1 = first administration, same day of the procedure; T2 = second administration, 24 hours after the procedure.

In order to enable future quality-of-life analyses comparing the radial and femoral accesses, patients underwent simple randomization for allocation to one type of access, using the REDCap system, soon after they agreed to participate in the study. A flowchart illustrating the randomization process is shown in Figure 5.

### Statistical analyses

Statistical analyses were conducted with the aid of the Statistical Package for the Social Sciences (v25.0), adopting a 5% significance level (p < 0.05). Data were tested for normality using the Shapiro-Wilk test. Differences in CS and EQL scores between T1 and T2 were tested using Wilcoxon’s nonparametric test.

Construct validity was assessed by estimating the interclass correlation between the scores obtained with each of the two questionnaires, using Spearman correlation coefficients. The internal consistency of the CS was estimated using Cronbach’s alpha coefficient, while reproducibility was verified using the Intraclass Correlation Coefficient (ICC) for the test-retest (T1 vs. T2).

## RESULTS

The Brazilian Portuguese version of the CS was administered to 62 patients of both sexes who had undergone diagnostic arteriography. Of these, 54.8% were men and 56.5% underwent femoral artery puncture. None of the patients needed their access changed and there were no failures during the arterial puncture procedures. None of the variables analyzed were normally distributed and nonparametric statistical tests were therefore used for all subsequent analyses.

### Linguistic assessment

The patients asked to evaluate the CS reported good comprehension of the items and the terms used. Just one participant reported difficulty understanding question 1, but did not specify what the problem was or make any suggestions for improvement. However, five patients considered that the first question took too long to read and one patient made the same observation in relation to items 3, 4, 5, and 7. None of the participants made any suggestions for terminological or conceptual improvements to the questionnaire.

### Analysis of differences between the two different questionnaire administration times

**Figure 6:**
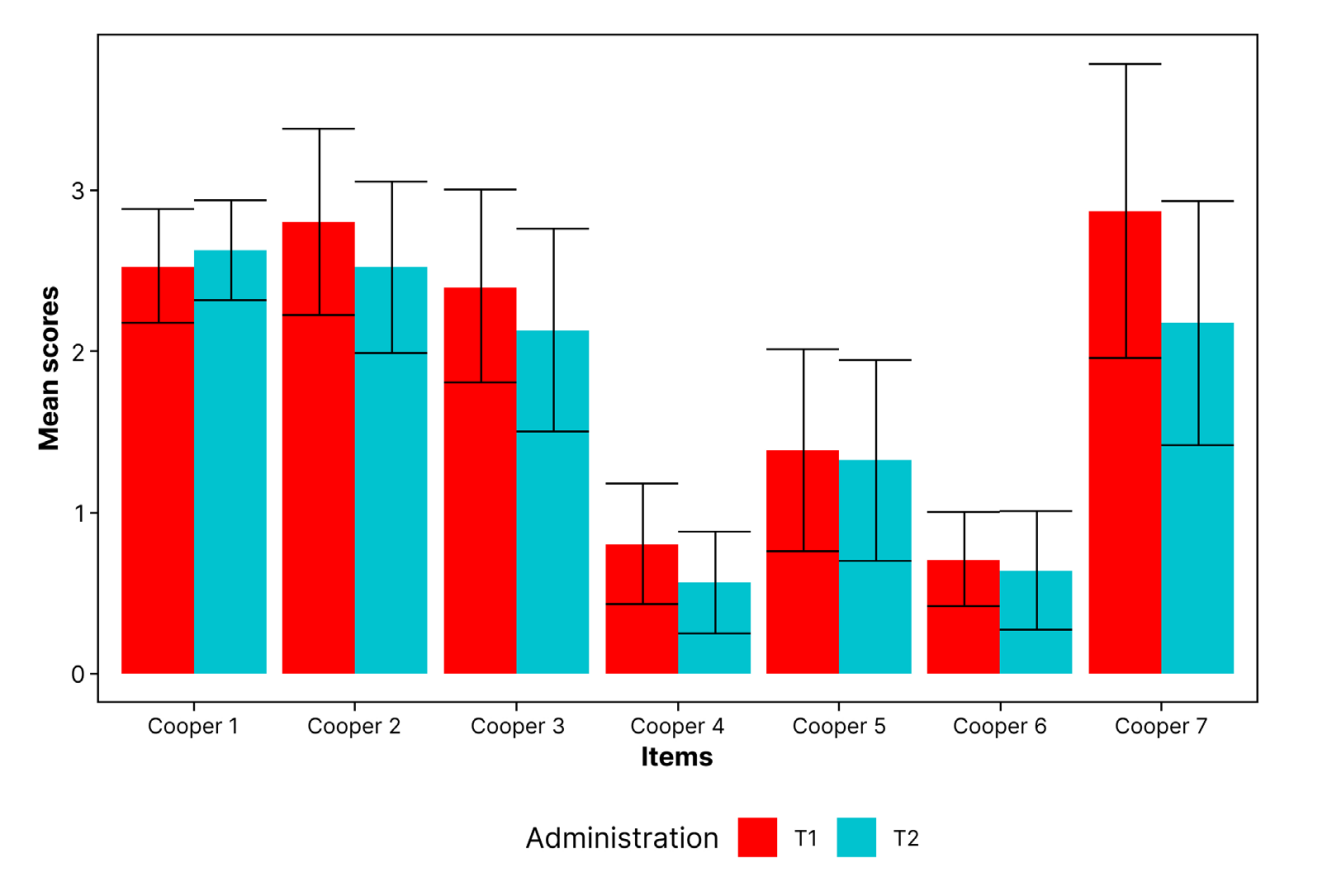
Graph comparing mean scores for the items on the Cooper Scale between T1 and T2 (n = 62). T1 = first administration; T2 = second administration.

Wilcoxon’s nonparametric test for repeated measures was used to compare mean values for numerical variables related to the CS and EQL questionnaires between the two times of administration (T1 and T2). The comparison between mean values for the CS questions at T1 and T2 is illustrated in Figure 6.

### Construct validation

Construct validity was assessed using the scores from T1 (n = 62), with the Spearman nonparametric correlation test (rho coefficient), since all variables had non-normal distributions. There was a significant correlation between CS item 7 and all the EQL items. All of these correlations were positive, except for the correlation with the last EQL item (“How good or bad is your health TODAY?”), which was negative. Significant positive correlations were also identified between CS item 2 and the EQL items related to the domains “Usual activities”, “Pain/Discomfort”, and “Anxiety/Depression” (p < 0.05) (Table 1).

**Table 1:** Spearman correlation coefficients (rho).

**Correlations**	**“How good or bad is your health today?” (T2)**	**CS 2 (T2)**	**CS 3 (T2)**	**CS 4 (T2)**	**CS 5 (T2)**	**CS 6 (T2)**	**CS 7 (T2)**
How good or bad is your health today? (T1)	0.706**	-0.197	0.012	0.015	0.059	-0.090	-0.210
CS 2 (T1)	0.026	0.474**	0.318*	0.077	-0.067	0.086	0.109
CS 3 (T1)	0	0.574**	0.727**	0.243	-0.043	0.347**	0.095
CS 4 (T1)	0.079	0.186	0.238	0.617**	0.261*	0.300*	0.103
CS 5 (T1)	0.132	0.208	0.197	0.194	0.589**	0.314*	0.529**
CS 6 (T1)	-0.194	0.243	0.285*	0.277*	0.247	0.612**	0.303*
CS 7 (T1)	-0.041	0.269*	0.014	-0.070	0.277*	0.190	0.737**

CS = Cooper Scale; T1 = time 1; T2 = time 2.

* p < 0.05.

** p < 0.01.

The values shown are Spearman coefficients (rho).

### Internal consistency and reproducibility

The internal consistency of the CS was assessed using the data from T1, returning a Cronbach’s Alpha of 0.519, indicating moderate internal consistency. Analysis of the column “Cronbach’s Alpha if the item is excluded” showed that all the items on the questionnaire made a positive contribution to its reliability. Exclusion of item 1 would result in a small increase in its Cronbach’s Alpha, to 0.535.

Test-retest reproducibility was verified by calculating the ICC for T1 against T2. Some of the CS items had excellent correlations, such as items 5 and 7 (ICC = 0.834; p < 0.001), whereas others had reasonable correlations, such as item 2 (ICC = 0.563; p < 0.001).

## DISCUSSION

Using instruments to assess quality of life is extremely important, particularly when the results can influence medical practice. As use of radial access for arteriography and angioplasties has increased, with very similar complication rates to femoral access, it has become very important to determine patients’ preferences to guide the choice of the best puncture site.^6,16^

In this study, international guidelines were followed for the cross-cultural adaptation and linguistic validation of the instrument, considering the cultural, structural, conceptual, and semantic aspects of all regions of Brazil.^10,17,18^ The translation and back-translation process was conducted with the support of qualified professional translators, in conjunction with the authors and a multidisciplinary committee made up of professionals with experience in hemodynamics. All of those involved, including the patients, rated the questionnaire as easy to answer and understand, with accessible vocabulary routinely used in the hemodynamics department.

The instrument’s good acceptability may in part be attributable to its concise structure – comprising just seven questions – and its use of few technical terms. Moreover, several of the terms used in the questionnaire are also used in the free and informed consent form, which is routinely administered at the institution, consolidating the patients’ prior experience with this vocabulary. While obtaining consent, professionals in the medical team also explained terms such as “puncture”, further facilitating their comprehension when the quality-of-life instrument was administered.

The internal consistency of the Brazilian version of the CS, as measured by Cronbach’s Alpha (α = 0.51), was considered moderate. This index assesses redundancy between questions and the instrument’s internal coherence, offering an estimate of whether all the items make a relevant contribution to the total score. Removal of item 1 would slightly raise the value of Cronbach’s alpha to 0.535. However, this item asks the patient about their preference between the two arterial puncture sites (radial or femoral), making it relevant to the questionnaire’s objectives. It is important to note that the CS necessarily comprises items with a certain degree of overlap, since it covers symptoms and limitations that frequently occur together after these procedures. As such, a certain amount of redundancy among its items is to be expected and is acceptable within the theoretical model underlying the instrument.

Test-retest reliability was assessed using ICC, demonstrating excellent reproducibility for the majority of questions (ICC > 0.750). Exceptions were items 2 (ICC = 0.563, considered reasonable) and 6 (ICC = 0.641, considered good). These weaker correlations can be explained by the characteristics of the items themselves: item 2 asks about general discomfort after the procedure and item 6 is about limitations affecting basic activities, such as eating and self-care. Both are conditions that tend to improve rapidly after the first 24 hours – the time that elapsed between the two administrations of the instrument –, especially after removal of the compressive dressing. Moreover, the reduction in the number of repeated administrations (test-retest) may have limited the statistical analysis of these variations. Originally, a third administration was planned (from 7 to 14 days post-procedure), but because of the COVID-19 pandemic and the institution’s prioritization of examinations for hospitalized patients with surgical referrals less than 48 hours after diagnosis, this third stage had to be eliminated. This reduced the number of assessments originally planned.

The instrument’s prospective responsiveness to change over time between repeated measures of a test was demonstrated in this study. It was observed that all of the CS variables had lower values at T1 than at T2, which is consistent with the expectation that symptoms should improve during the first 24 hours after arterial puncture. There was a statistically significant difference in CS item 3 values (p < 0.05), which underscores the instrument’s sensitivity for detecting changes in the patient’s status. These findings can be attributed to removal of the compressive dressing by T2, which makes a considerable contribution to increased patient comfort – irrespective of the access route used (femoral or radial).

If an instrument’s measurements are not comparable and its scores are not correlated over short periods of time, it is probable that such measurements are indicative of errors. Therefore, the instrument may not reflect the differences between groups, compromising its validity.^19^ In the present study, the CS proved to be a good instrument for detecting changes in quality of life after arterial puncture, including over short periods of time.

With regard to construct validity, the Brazilian Portuguese version of the CS was significantly correlated with the EQL, as validated with Spearman coefficients. Item 7 on the CS was positively correlated with all the questions on the EQL, except the last item, with which it had a positive correlation. This result was to be expected, since higher scores for the last item of the EQL indicate better quality of life, whereas higher values on the CS indicate worse quality of life.

Moreover, item 2 on the CS was significantly correlated with the domains “Usual activities” and “Anxiety/Depression” on the EQL questionnaire. In contrast, the other CS questions had no statistically significant correlations with the EQL, which may be because of the fact that it assesses more specific aspects of the puncture site, rather than the general state of health of the entire body, as is the case of items 2 and 7.

To date, there were no specific questionnaires available for Brazil to assess quality of life after arterial puncture, or studies focused on diagnostic arteriography in patients with peripheral arterial disease. Compared with other instruments that have been adapted for the Brazilian context, CS is the only questionnaire created specifically to assess the effects of arterial puncture,^6^ in contrast with generic quality-of-life questionnaires. The CS comprises a series of questions specifically related to the procedure, which assess aspects such as general discomfort, back pain, ability to go to the bathroom, to feed oneself, to care for oneself, and to walk, on a visual scale from 0 to 10. The CS is easy to understand and can be administered by any healthcare professional.

This type of instrument can make a significant contribution to making patients feel comfortable, especially those undergoing invasive examinations via femoral access, for which patients must be in bed and which causes significant discomfort during the period that the puncture site is subjected to manual compression.

Considering the Brazilian setting, in which arteriography is still widely used in a great many cases and is considered the gold standard for diagnosis of lower limb arterial disease,^17^ validation of the CS should facilitate identification of patients’ access preference (femoral or radial) in a population that has not been studied previously.

The international literature contains few questionnaires developed and validated specifically focused on quality of life after arterial puncture. The Cooper scale is one of the few specific instruments for this procedure.^14^ Therefore, using a generic instrument such as the EQL, which assesses quality of life in general, it was to be expected that all of the items would have significant correlations.

While conducting the literature review for this study, no instruments were found that were specifically developed for arterial puncture and had been translated to Brazilian Portuguese and validated. As such, this study constitutes a unique contribution in the Brazilian setting.

Limitations of this study include enrollment of both outpatients and hospitalized patients, rather than restricting the sample to outpatients. This decision was taken because of a change in the hospital’s care profile during the COVID-19 pandemic and could have introduced bias to the study design, since these two classes of patients may have different perceptions of their quality of life after arterial puncture. Nevertheless, comparing these two groups was never part of the study objectives, which were limited to translation and validation of the questionnaire.

Another limitation is related to the fact that the study was conducted at a quaternary hospital, which could limit its external validity. Additionally, the original study protocol stipulated a third administration of the instrument (from 7 to 14 days after T1), but this stage became unfeasible because of the large number of losses of outpatients follow-up, attributable to the COVID-19 pandemic, and because hospitalized patients underwent definitive surgical treatment. However, this change did not introduce bias, since these losses would only have affected the quality-of-life assessment, which was not part of the study objective.

While construct validity was only moderate, the results for reproducibility (test-retest) were highly satisfactory, supporting the reliability of the Brazilian Portuguese version of the CS. This questionnaire can therefore be used to assess quality of life in Brazilian patients who need arterial puncture, including for coronary angiography and percutaneous coronary procedures that utilize a radial access – which had never been assessed in the Brazilian population, since, until now, there were no questionnaires available that had been translated and validated in our language.

## CONCLUSIONS

This study conducted the translation, cross-cultural adaptation, and psychometric validation of the CS in Brazilian Portuguese, with the objective of making an instrument available that could be used to assess the quality of life of patients after arterial puncture. The process adhered rigorously to international guidelines for linguistic validation and included a cognitive assessment in the target population.

The results demonstrated that the Brazilian version of the CS had satisfactory reliability, assessed using tests of internal consistency and reproducibility (test-retest), and moderate construct validity, with significant correlations with the EQL instrument. These findings confirm that the instrument is adequate for clinical use and in patients who undergo diagnostic arteriography.

Additional studies with the validated Brazilian Portuguese version of the CS should be conducted in different specialties, such as vascular surgery, interventional radiology, and cardiology, in order to improve understanding of the impact of arterial puncture on quality of life among different subgroups of patients.
