# Supplementary material for: Translation and validation into Brazilian Portuguese of the Post-Catheterization Questionnaire, or Cooper Scale, for post-puncture quality of life analysis in diagnostic arteriography of the lower limbs
Source: J Vasc Bras. 2026 Mar 16;24:e20240065. doi: 10.1590/1677-5449.202400652 (PMC13007340; doi:10.1590/1677-5449.202400652)
Supplement: Translation

# Translation: Tradução e validação para o português brasileiro do Post-Catheterization Questionnaire, ou Escala de Cooper, para análise da qualidade de vida após punção arterial em arteriografia diagnóstica dos membros inferiores

**DOI:** 10.1590/1677-5449.202400651

**Authors:** Igor Calixto Novais Dias, Marcelo Passos Teivelis, Gabriel Grizzo Cucato, Felipe Nasser, Marcelo Calil Burihan, Henrique Jorge Guedes

**Affiliations:** 1 Faculdade Santa Marcelina, São Paulo, SP, Brasil.; 2 Faculdade Israelita de Ciências da Saúde Albert Einstein – FICSAE, São Paulo, SP, Brasil.; 3 Universidade Federal de São Paulo – UNIFESP, São Paulo, SP, Brasil.

**Keywords:** Escala de Cooper, questionários, validação, radial, arteriografia

## Abstract

**Contexto:**

A Escala de Cooper é um questionário originalmente desenvolvido em língua inglesa para avaliar a qualidade de vida após punção arterial em procedimentos como arteriografia e angioplastia. A escala analisa, especificamente, a preferência do paciente entre os acessos radial e femoral.

**Objetivos:**

Traduzir e validar a Escala de Cooper para o português brasileiro.

**Métodos:**

O processo de tradução seguiu diretrizes internacionais de validação linguística e foi conduzido com 62 pacientes portadores de doença arterial obstrutiva periférica submetidos à arteriografia diagnóstica em um hospital quaternário de São Paulo. A confiabilidade foi avaliada por meio de testes de reprodutibilidade (teste-reteste com intervalo de 24 horas) e de consistência interna. A validade de construto foi analisada a partir da correlação entre os itens da Escala de Cooper e os do EUROQOL 5D-5L, o qual também foi respondido pelos 62 pacientes.

**Resultados:**

A Escala de Cooper apresentou consistência interna moderada e alta confiabilidade no teste-reteste, com coeficientes de correlação intraclasse variando de 0,563 a 0,834 (p < 0,001). Foram observadas correlações significativas entre os itens da Escala de Cooper e os do EQ-5D-5L.

**Conclusões:**

A Escala de Cooper foi traduzida e validada para o português do Brasil, demonstrando adequação psicométrica e confiabilidade para aplicação em pacientes submetidos à punção arterial.

## INTRODUÇÃO

A angiografia por subtração digital é considerada uma das principais técnicas para obtenção de imagens vasculares^1,2^ , especialmente dos membros inferiores. A escolha adequada do sítio de punção é fundamental para reduzir a incidência de complicações e otimizar o tempo do procedimento. O acesso ideal deve ser de fácil localização, com base em referências anatômicas ou na palpação do pulso, podendo também contar com o auxílio de métodos como a radioscopia e o eco-Doppler^2^ . Historicamente, o acesso femoral tem sido o mais utilizado nas arteriografias de membros inferiores, chegando a representar mais de 80% dos casos de angiografias cardíacas nos EUA nos anos 2000^2^ . Entretanto, esse tipo de punção exige que os pacientes permaneçam acamados e causa desconforto significativo durante a compressão da área puncionada, principalmente nas primeiras 24 horas após o exame.

Com a evolução tecnológica, os dispositivos utilizados nos procedimentos tornaram-se menores, com melhor navegabilidade e torque, permitindo o uso da artéria radial como nova via de acesso. Esse acesso apresenta índices de complicações semelhantes e maior qualidade de vida para os pacientes após o procedimento^3-5^ . No Japão, a artéria radial chega a representar aproximadamente 75% dos casos de intervenção coronária^4^ . Apesar de seu menor calibre e da maior dificuldade técnica para a punção, o acesso radial oferece vantagens como maior conforto ao paciente, facilidade no monitoramento de complicações imediatas, deambulação precoce e menor tempo de internação^6^ .

Embora a artéria radial também possa ser utilizada em procedimentos voltados a outros territórios arteriais, como membros inferiores com doença arterial oclusiva periférica, ainda não há evidências de que pacientes com essa condição prefiram esse tipo de acesso, como ocorre com aqueles com doença coronariana. Dessa forma, questionários de qualidade de vida podem ajudar a avaliar essa preferência.

Considerando o cenário nacional, em que ainda se realiza um grande número de exames invasivos, como arteriografias dos membros inferiores e aortografias, a utilização de instrumentos específicos e validados em português do Brasil pode auxiliar na escolha do melhor acesso arterial nesse subgrupo de pacientes.

Assim, este estudo teve como objetivo traduzir e validar o questionário Post-Catheterization Questionnaire, também conhecido como Escala de Cooper (EC), para o português brasileiro. Esse instrumento, descrito inicialmente por Cooper et al.^6^ , avalia a qualidade de vida após punção arterial, contribuindo para a definição do acesso ideal para os diferentes grupos de pacientes que necessitam de exames invasivos como a arteriografia diagnóstica.

## MÉTODOS

O estudo foi realizado no Hospital Israelita Albert Einstein (HIAE), em São Paulo, com coparticipação do Hospital Santa Marcelina (HSM). O projeto foi registrado na Plataforma Brasil (HIAE Certificado de Apresentação de Apreciação Ética (CAAE): 02763518.9.0000.0071; HSM CAAE: 02763518.9.3001.0066) e aprovado pelos Comitês de Ética em Pesquisa do HIAE (parecer nº 3.241.955) e do HSM (parecer nº 3.482.845/2019).

### Procedimentos de tradução e adaptação cultural

Seguindo protocolos previamente descritos^7-11^ , a EC foi submetida a um processo de tradução e adaptação transcultural, utilizando o método de tradução reversa^7^ . Inicialmente, a tradução foi realizada por um profissional independente, brasileiro nativo e fluente em inglês. O tradutor não recebeu informações sobre o objetivo do estudo e foi orientado a realizar a tradução semântica, e não apenas literal.

Posteriormente, a versão traduzida foi revisada, junto aos autores, por um comitê multidisciplinar composto por médicos-cirurgiões vasculares, enfermeiros da hemodinâmica e cardiologistas, todos com experiência em arteriografia diagnóstica. Essa equipe avaliou cada item do questionário e sugeriu ajustes para aprimoramento da linguagem, quando pertinente. Nessa etapa, os autores, em conjunto com o comitê e com o auxílio do tradutor, também avaliaram possíveis variações linguísticas regionais do Brasil. Com base nessas contribuições, foi elaborada uma segunda versão do instrumento.

Na sequência, foi realizada a retrotradução por um tradutor profissional, nativo da língua inglesa e fluente em português brasileiro. Esse profissional não teve acesso ao instrumento original. A versão retrotraduzida foi comparada à versão original em inglês pelo mesmo comitê multidisciplinar e pelos autores, resultando em uma versão final consensual em português, com vocabulário simples e direto. A versão original da EC e sua tradução final estão apresentadas nas Figuras 1 e 2, respectivamente.

**Figura 1:** Escala de Cooper original.

**Figura 2:** Escala de Cooper – versão final em português do Brasil.

### Validação linguística

A validação linguística é o processo a partir do qual um instrumento é testado entre representantes da população-alvo para determinar se os entrevistados entendem o questionário traduzido da mesma forma que o original seria compreendido. De acordo com protocolos internacionais^8-11^ , inicialmente é realizado um teste-piloto com três a cinco participantes, enquanto as demais etapas requerem de sete a dez participantes por questão. Como a EC possui sete questões, seria necessário um mínimo de 49 pacientes.

Dessa forma, foi conduzido um teste-piloto com cinco pacientes, que avaliaram a clareza do questionário e dos termos utilizados, além de fazerem sugestões de melhorias, quando pertinente. Uma reanálise foi realizada pelos autores com base nas sugestões apresentadas pelos participantes. Optou-se por manter a análise cognitiva nos primeiros 62 pacientes incluídos. O fluxo do processo de validação linguística pode ser visualizado na Figura 3.

**Figura 3:** Fluxo de validação linguística transcultural do instrumento Escala de Cooper do inglês (idioma original) para o português brasileiro (idioma-alvo).

### Validação psicométrica

A validação psicométrica foi conduzida com os mesmos 62 pacientes, que responderam à EC em dois momentos: imediatamente após a retirada do introdutor (M1) e 24 horas após o procedimento (M2). Entrevistadores diferentes aplicaram o questionário em cada momento. Embora o protocolo original previsse uma terceira aplicação entre 7 e 14 dias após o M1, essa etapa foi suspensa devido a perdas de seguimento, atribuídas à pandemia de covid-19 e à realização precoce de procedimentos cirúrgicos definitivos nos pacientes internados.

A confiabilidade da EC foi avaliada por meio de testes de reprodutibilidade (teste-reteste) e consistência interna^12,13^ . A validade do construto foi testada por meio da comparação da EC com o questionário EUROQOL 5D-5L (EQL), que também foi respondido pelos mesmos 62 pacientes. O EQL foi escolhido por ser um questionário de qualidade de vida de fácil aplicabilidade e já validado para o português brasileiro^10^ . O fluxo da validação pode ser visualizado na Figura 4.

**Figura 4:** Fluxo de validação linguística e psicométrica da Escala de Cooper e suas respectivas amostras dos participantes do estudo.

### População e critérios de inclusão/exclusão

Foram incluídos pacientes com indicação de arteriografia diagnóstica de membros inferiores, atendidos em hospital quaternário entre 2020 e 2023. Todos receberam e assinaram o Termo de Consentimento Livre e Esclarecido (TCLE) para o procedimento, previamente padronizado pela instituição, o qual continha os termos técnicos utilizados no questionário e que também foram explicados pela equipe médica durante a aplicação do TCLE. Devido à pandemia de covid-19, o desenho original do estudo, que incluiria apenas pacientes ambulatoriais, foi abandonado, sendo incluídos também pacientes internados. Todos os pacientes concordaram em participar do estudo.

Os critérios de exclusão foram pacientes com ausência de onda no teste de Barbeau tipo 4^4^ , falha na tentativa de cateterização de qualquer via de acesso, presença de oclusão da artéria ulnar ou artéria femoral, coagulopatias conhecidas, artéria radial com diâmetro inferior a 2 mm e pacientes analfabetos.

Todos os exames foram realizados por médicos com experiência em ambos os acessos, seguindo protocolos internacionais^14,15^ . A hemostasia foi obtida com compressão manual por 15 minutos, seguida de curativo compressivo por 6 horas na artéria femoral ou 4 horas na artéria radial.

### Randomização

Para viabilizar futuras análises comparativas de qualidade de vida entre os acessos radial e femoral, os pacientes foram randomizados de forma simples para cada tipo de acesso, utilizando o sistema REDCap, logo após aceitarem participar do estudo. O fluxo de randomização está disponível na Figura 5.

**Figura 5:** Fluxo de inclusão dos pacientes para análise dos dados e tempos de aplicação dos questionários. TCLE = Termo de Consentimento Livre e Esclarecido; EQL = EUROQOL 5D-5L; EC = Escala de Cooper; t1 = primeira administração, mesmo dia do procedimento; t2 =segunda administração, 24 horas após o procedimento.

### Análises estatísticas

As análises estatísticas foram realizadas com o *software* Statistical Package for the Social Sciences (v25.0), adotando-se um nível de significância de 5% (p < 0,05). Os dados foram avaliados quanto à normalidade por meio do teste de Shapiro-Wilk. Para verificar diferenças nas pontuações da EC e do EQL entre M1 e M2, foi utilizando o teste não paramétrico de Wilcoxon.

A validade do construto foi avaliada por meio da correlação interclasse entre os escores obtidos nos dois questionários, utilizando o coeficiente de correlação de Spearman. A consistência interna da EC foi determinada pelo coeficiente alfa de Cronbach, enquanto a reprodutibilidade foi verificada através do coeficiente de correlação intraclasse (CCI) no teste-reteste entre M1 e M2.

## RESULTADOS

A versão em português brasileiro da EC foi administrada a 62 pacientes, de ambos os sexos, submetidos a arteriografia diagnóstica. Desses, 54,8% eram homens, e 56,5% foram submetidos a punção da artéria femoral. Não foi necessária a troca de acesso, e não ocorreram falhas no procedimento de punção arterial. Nenhuma das variáveis analisadas apresentou distribuição normal e, portanto, foram utilizados testes estatísticos não paramétricos nas análises subsequentes.

### Avaliação linguística

Na avaliação da EC, os pacientes relataram boa compreensão dos itens e dos termos empregados. Apenas um participante informou dificuldade para compreender a questão 1, mas não especificou o problema e nem apresentou sugestões. Além disso, cinco pacientes consideraram a leitura da primeira pergunta muito extensa, e um paciente fez a mesma observação em relação às perguntas 3, 4, 5 e 7. Nenhum participante fez sugestões de melhorias terminológicas ou conceituais para o questionário.

### Análise das diferenças entre os tempos

Para comparar os valores médios das variáveis numéricas relacionadas aos questionários EC e EQL entre os dois tempos de aplicação (M1 e M2), foi utilizado o teste não paramétrico de Wilcoxon para medidas repetidas. A comparação entre os valores médios das questões da EC nos dois tempos (M1 e M2) está apresentada na Figura 6.

**Figura 6:** Representação gráfica da comparação dos valores médios das questões da Escala de Cooper entre M1 e M2 (n = 62). M1 = momento 1; M2 = momento 2.

### Validação do construto

A validade do construto foi avaliada com base nos escores obtidos em M1 (n = 62), utilizando-se o teste não paramétrico de correlação de Spearman (coeficiente rho), uma vez que todas as variáveis apresentaram distribuição não normal. Observou-se correlação significativa entre a questão 7 da EC e todas as questões do EQL. Todas essas correlações foram positivas, exceto a última questão do EQL (“Quão boa ou ruim a sua saúde está hoje?”), que apresentou correlação negativa. Também foram identificadas correlações positivas significativas entre a questão 2 da EC e as questões relacionadas aos domínios “Atividades habituais”, “Dor/Mal-estar” e “Ansiedade/Depressão” do EQL (p < 0,05) (Tabela 1).

**Tabela 1:** Valores do coeficiente de Spearman (rho).

**Correlações**	**“Quão boa ou ruim a sua saúde está hoje?” (M2)**	**EC 2 (M2)**	**EC 3 (M2)**	**EC 4 (M2)**	**EC 5 (M2)**	**EC 6 (M2)**	**EC 7 (M2)**
Quão boa ou ruim a sua saúde está hoje? (M1)	0,706**	-0,197	0,012	0,015	0,059	-0,090	-0,210
EC 2 (M1)	0,026	0,474**	0,318*	0,077	-0,067	0,086	0,109
EC 3 (M1)	0	0,574**	0,727**	0,243	-0,043	0,347**	0,095
EC 4 (M1)	0,079	0,186	0,238	0,617**	0,261*	0,300*	0,103
EC 5 (M1)	0,132	0,208	0,197	0,194	0,589**	0,314*	0,529**
EC 6 (M1)	-0,194	0,243	0,285*	0,277*	0,247	0,612**	0,303*
EC 7 (M1)	-0,041	0,269*	0,014	-0,070	0,277*	0,190	0,737**

EC = Escala de Cooper; M1 = momento 1; M2 = momento 2.

* p < 0,05.

** p < 0,01.

Os valores apresentados correspondem ao coeficiente de Spearman (rho).

### Consistência interna e reprodutibilidade

A consistência interna da EC, avaliada com base nos dados de M1, apresentou um alfa de Cronbach de 0,519, indicando consistência interna moderada. A análise da coluna “alfa de Cronbach se o item for excluído” mostrou que todos os itens do questionário contribuíram positivamente para a confiabilidade da escala. A exclusão da questão 1 resultaria em um pequeno aumento do alfa de Cronbach para 0,535.

A reprodutibilidade do teste-reteste foi verificada por meio do CCI, calculado entre M1 e M2. Algumas questões da EC apresentaram excelente correlação, como as questões 5 e 7 (CCI = 0,834; p < 0,001), enquanto outras demonstraram correlação razoável, como a questão 2 (CCI = 0,563; p < 0,001).

## DISCUSSÃO

O uso de instrumentos que avaliam a qualidade de vida é de extrema importância, principalmente quando os resultados podem influenciar a conduta médica. Com o aumento do uso do acesso radial para arteriografias e angioplastias, com uma taxa de complicações muito similar ao acesso femoral, definir a preferência dos pacientes torna-se fundamental para guiar a escolha do melhor sítio de punção^6,16^ .

Neste estudo, foram seguidas as diretrizes internacionais para adaptação transcultural e validação linguística do instrumento, considerando os aspectos culturais, estruturais, conceituais e semânticos de todas as regiões do Brasil^10,17,18^ . O processo de tradução e retrotradução foi realizado com o apoio de tradutores profissionais formados em Letras, em conjunto com os autores e um comitê multidisciplinar composto por profissionais experientes da área de hemodinâmica. Todos os envolvidos, incluindo os pacientes, avaliaram o questionário como de fácil aplicação e compreensão, com uso de vocabulário acessível e usado rotineiramente no setor de hemodinâmica.

A boa aceitação do instrumento pode ser atribuída, em parte, à sua estrutura concisa – composta por apenas sete perguntas – e à utilização de poucos termos técnicos. Ademais, muitos dos termos utilizados no questionário também são utilizados no TCLE, aplicado de forma regular pela instituição, o que reforça o contato prévio dos pacientes com esse vocabulário. Durante a obtenção do consentimento, profissionais da equipe médica também esclarecem expressões como “punção”, facilitando ainda mais a compreensão durante a aplicação do instrumento.

A consistência interna da versão brasileira da EC, medida pelo alfa de Cronbach (α = 0,51), foi considerada moderada. Esse índice avalia a redundância entre as questões e a coerência interna do instrumento, permitindo estimar se todos os itens contribuem de maneira relevante para o escore total. A exclusão da questão 1 elevaria ligeiramente o valor do alfa para 0,535; no entanto, essa questão está relacionada à preferência do paciente entre os dois tipos de punção arterial (radial ou femoral), o que a torna relevante para os objetivos do questionário. É importante destacar que a EC, por sua própria natureza, possui itens com algum grau de sobreposição, visto que trata de sintomas e limitações que frequentemente ocorrem em conjunto após o procedimento. Portanto, a presença de certa redundância entre as questões é esperada e aceitável dentro do modelo teórico do instrumento.

A confiabilidade teste-reteste, avaliada por meio do CCI, demonstrou excelente reprodutibilidade para a maioria das questões (CCI > 0,750). Exceções foram observadas nas questões 2 (CCI = 0,563, considerada razoável) e 6 (CCI = 0,641, considerada boa). Essas correlações menores podem ser explicadas pelas características dos próprios itens: a questão 2 aborda o desconforto geral após o procedimento, enquanto a questão 6 trata de limitações em atividades básicas, como alimentação e autocuidado. Ambas refletem condições que tendem a melhorar rapidamente após as primeiras 24 horas – intervalo entre os dois momentos de aplicação do instrumento –, especialmente após a retirada do curativo compressivo. Além disso, o número reduzido de aplicações repetidas (teste-reteste) pode ter limitado a análise estatística dessas variações. Originalmente, previa-se a realização de uma terceira aplicação (entre 7 e 14 dias), mas, devido à pandemia de covid-19 e à priorização institucional de exames em pacientes internados com encaminhamento cirúrgico em menos de 48 horas após o diagnóstico, essa etapa teve que ser suprimida. Esse contexto limitou o número de avaliações originalmente idealizado.

A responsividade longitudinal do instrumento, que avalia se há mudanças ao longo do tempo entre as medidas repetidas do teste, foi demonstrada neste estudo. Observou-se que todas as variáveis da EC apresentaram valores médios inferiores em M1 em relação ao M2, o que é consistente com a expectativa de melhora dos sintomas nas primeiras 24 horas após a punção arterial. Houve diferença estatisticamente significativa na questão 3 da EC (p < 0,05), o que reforça a sensibilidade do instrumento para captar alterações no estado do paciente. Esses achados podem ser atribuídos à remoção do curativo compressivo em M2, fator que contribui substancialmente para o aumento do conforto do paciente – independentemente da via de acesso utilizada (femoral ou radial).

Se as medidas de um instrumento não forem comparáveis e os escores não apresentarem correlações em pequenos intervalos de tempo, é provável que essas medidas indiquem erros. Portanto, o instrumento pode não refletir as diferenças entre os grupos, comprometendo sua validade^19^ . No presente estudo, a EC se mostrou um bom instrumento para detectar mudanças na qualidade de vida após punção arterial, inclusive em pequenos intervalos de tempo.

No que se refere à validade do construto, a versão em português brasileiro da EC apresentou correlações significativas com o EQL, validadas pelo coeficiente de Spearman. A questão 7 da EC demonstrou correlação positiva com todas as questões do EQL, exceto a última, com a qual demonstrou correlação negativa. Esse resultado era esperado, uma vez que escores mais altos na última questão do EQL indicam melhor qualidade de vida, enquanto, na EC, valores mais elevados indicam pior qualidade de vida.

Além disso, a questão 2 da EC apresentou correlações significativas com os domínios “Atividade habituais”, “Dor/Mal-estar” e “Ansiedade/Depressão” no questionário EQL. Por outro lado, as demais questões da EC não apresentaram correlações estatisticamente significativas com o EQL, o que pode ser explicado pelo fato de avaliarem aspectos mais específicos da região puncionada, em vez de condições gerais do corpo, como ocorre nas questões 2 e 7.

Até o momento, não havia no Brasil um questionário específico para avaliar a qualidade de vida após a punção arterial, tampouco estudos voltados às arteriografias diagnósticas em pacientes com doença arterial periférica. Em comparação com outros instrumentos adaptados para o contexto brasileiro, a EC é o único questionário criado especificamente para avaliar os efeitos da punção arterial^6^ , diferentemente de questionários genéricos de qualidade de vida. A EC é composta por uma série de perguntas relacionadas especificamente ao procedimento, que avaliam aspectos como desconforto geral, dor nas costas, capacidade de usar o banheiro, de se alimentar, de cuidar de si próprio e de andar, por meio de uma escala visual de 0 a 10. A EC é de fácil compreensão e pode ser aplicada por qualquer profissional de saúde.

Esse tipo de instrumento pode contribuir significativamente para o acolhimento dos pacientes, especialmente em exames invasivos realizados por via femoral, que exigem que os pacientes fiquem acamados e geram desconforto significativo durante o período de compressão manual da área puncionada.

Considerando o cenário nacional, em que a arteriografia continua sendo amplamente utilizada em um grande número de casos e é considerada o padrão-ouro para o diagnóstico da doença arterial dos membros inferiores^17^ , a validação da EC poderá auxiliar na identificação da preferência pela via de acesso (femoral ou radial) em uma população ainda não estudada.

Questionários voltados especificamente à avaliação da qualidade de vida após punção arterial são pouco desenvolvidos e validados na literatura internacional. A EC é um dos raros instrumentos direcionados à avaliação de um procedimento específico^14^ . Portanto, ao empregar um instrumento genérico como o EQL, que avalia de forma geral a qualidade de vida, já se esperava que nem todas as questões apresentassem correlações significativas.

Durante a revisão da literatura realizada neste estudo, não foram identificados instrumentos específicos para a avaliação da punção arterial que tivessem sido traduzidos e validados para o português do Brasil com o mesmo objetivo. Assim, este trabalho representa uma contribuição inédita para o cenário nacional.

Entre as limitações do estudo, destaca-se a inclusão de pacientes tanto internados quanto ambulatoriais, em vez de apenas ambulatoriais. Essa decisão decorreu de uma mudança no perfil assistencial do hospital durante a pandemia de covid-19, o que pode ter introduzido viés no desenho do estudo, uma vez que esses dois grupos de pacientes podem apresentar percepções diferentes em relação à qualidade de vida após a punção arterial. No entanto, essa comparação entre grupos não fazia parte dos objetivos do presente estudo, que se restringiu à tradução e validação do questionário.

Outra limitação refere-se ao fato de o estudo ter sido realizado em um hospital quaternário, o que pode limitar sua validade externa. Além disso, embora o protocolo original previsse uma terceira aplicação do instrumento (entre 7 e 14 dias após M1), essa etapa foi inviabilizada devido ao grande número de perdas de seguimento de pacientes ambulatoriais, atribuído à pandemia de covid-19 e à realização de tratamento cirúrgico definitivo nos pacientes internados. No entanto, essa alteração não se traduziu em um viés, pois tais perdas afetariam apenas a avaliação da qualidade de vida, que não era objeto de estudo.

Apesar de a validade de construto ter sido apenas moderada, os resultados de reprodutibilidade (teste-reteste) foram muito satisfatórios, reforçando a confiabilidade da versão em português brasileiro da EC. Esse questionário, portanto, pode ser utilizado para avaliar a qualidade de vida em pacientes brasileiros com necessidade de punções arteriais, incluindo angiografia coronária e procedimentos coronarianos percutâneos que utilizam a via radial – os quais nunca foram avaliados na população brasileira, uma vez que, até então, não havia um questionário traduzido e validado para o nosso idioma.

## CONCLUSÃO

Este estudo realizou a tradução, a adaptação transcultural e a validação psicométrica da EC para o português do Brasil, com o objetivo de disponibilizar um instrumento capaz de avaliar a qualidade de vida de pacientes após punções arteriais. O processo seguiu rigorosamente as diretrizes internacionais de validação linguística e incluiu avaliação cognitiva junto à população-alvo.

Os resultados demonstraram que a versão brasileira da EC apresentou confiabilidade satisfatória, avaliada por meio dos testes de consistência interna e reprodutibilidade (teste-reteste), além de validade de construto moderada, com correlações significativas com o instrumento EQL. Esses achados confirmam a adequação do instrumento para uso clínico e em pesquisas com pacientes submetidos à arteriografia diagnóstica.

Novos estudos com a versão validada da EC para o português brasileiro devem ser conduzidos em diferentes áreas, como cirurgia vascular, radiologia intervencionista e cardiologia, a fim de melhorar o entendimento do impacto da punção arterial na qualidade de vida de diferentes subgrupos de pacientes.
